# Morphometric study of the two fused primary ossification centers of the clavicle in the human fetus

**DOI:** 10.1007/s00276-016-1640-y

**Published:** 2016-02-09

**Authors:** Mariusz Baumgart, Marcin Wiśniewski, Magdalena Grzonkowska, Mateusz Badura, Małgorzata Dombek, Bogdan Małkowski, Michał Szpinda

**Affiliations:** 1 Department of Normal Anatomy, The Ludwik Rydygier Collegium Medicum in Bydgoszcz, The Nicolaus Copernicus University in Toruń, Łukasiewicza 1 Street, 85-821 Bydgoszcz, Poland; 2 Department of Positron Emission Tomography and Molecular Imaging, The Ludwik Rydygier Collegium Medicum in Bydgoszcz, The Nicolaus Copernicus University in Toruń, Łukasiewicza 1 Street, 85-821 Bydgoszcz, Poland

**Keywords:** Clavicle, Ossification center, Human fetus, Digital image analysis, CT examination, Regression analysis

## Abstract

**Purposes:**

A satisfactory understanding of the clavicle development may be contributing to both the diagnosis of its congenital defects and prevention of perinatal damage to the shoulder girdle. This study was carried out to examine the transverse and sagittal diameters, cross-sectional area and volume of the two fused primary ossification centers of the clavicle.

**Methods:**

Using the methods of CT, digital-image analysis and statistics, the size for two fused primary ossification centers of the clavicle in 42 spontaneously aborted human fetuses at ages of 18–30 weeks was studied.

**Results:**

Without any male–female and right-left significant differences, the best fit growth models for two fused primary ossification centers of the clavicle were as follows: *y* = −31.373 + 15.243 × ln(age) ± 1.424 (*R*^2^ = 0.74) for transverse diameter, *y* = −7.945 + 3.225 × ln(age) ± 0.262 (*R*^2^ = 0.78), *y* = −4.503 + 2.007 × ln(age) ± 0.218 (*R*^2^ = 0.68), and *y* = −4.860 + 2.117 × ln(age) ± 0.200 (*R*^2^ = 0.73) for sagittal diameters of the lateral, middle and medial ends respectively, *y* = −31.390 + 2.432 × age ± 4.599 (*R*^2^ = 0.78) for cross-sectional area, and *y* = 28.161 + 0.00017 × (age)^4^ ± 15.357 (*R*^2^ = 0.83) for volume.

**Conclusions:**

With no sex and laterality differences, the fused primary ossification centers of the clavicle grow logarithmically in both transverse and sagittal diameters, linearly in cross-sectional area, and fourth-degree polynomially in volume. Our normative quantitative findings may be conducive in monitoring normal fetal growth and screening for inherited faults and anomalies of the clavicle in European human fetuses.

## Introduction

A satisfactory understanding of the clavicle development may be conducive in both the diagnosis of its congenital defects and prevention of perinatal damage to the shoulder girdle [3, 12, 24, 31, 32]. Primary ossification in the human embryo commences just between weeks 5 and 6 in a condensed rod of mesenchyme of the shaft of the clavicle [5, 9, 25]. On the 45th day its primary medial and lateral intramembranous spots of ossification blend between the middle and lateral thirds of the bone [15]. Since both the shaft of the clavicle and most cranial bones develop in membrane, their concurred defects of ossification result in hereditary cleidocranial dysplasia [32]. As a result of both spontaneous genetic mutations or disorders in embryogenesis, defects of the shoulder girdle mostly appear up to the 7th week of intrauterine life, and can be recognized by ultrasound in fetuses from week 18 onwards. Common perinatal damages to the clavicle mainly include its fracture with incidence of 1.6 %, particularly at delivery in a shoulder presentation [12].

To date however, neither numerical data nor nomograms for the fused primary ossification centers of the clavicle have been assessed in the human fetus. To our opinion, the problem of the quantitative growth of the clavicle should be opened on adequate material. Therefore, the objectives of the present study were to:

- perform morphometric analysis of linear, planar and spatial parameters of the fused primary ossification centers of the clavicle to establish a range of their normative values,
- examine possible sex differences for all the measured parameters,
- develop explicit growth dynamics for all studied parameters, expressed by best fit mathematical functions.

## Materials and methods

The study material was composed of 42 European human fetuses of both sexes, 21 males and 21 females, at the age range of 18–30 weeks of gestation, derived from either spontaneous miscarriages or premature births. Since neither conspicuous internal nor external anatomical malformations were found on macroscopic examination, the entire sample could be considered normal. In addition, as correlation between the gestational age based on the crown-rump length and that calculated by the last menstruation attained the value *R* = 0.98 (*P* < 0.001), the specimens under examination could not suffer from growth retardation. The sample came from a large collection gathered before the year 2000 at Department of Normal Anatomy of our university. The study was sanctioned by the Ethics Committee of Ludwik Rydygier Collegium Medicum in Bydgoszcz (KB 275/2011). Fetal ages were established on the specimen’s crown-rump length. Table 1 presents the gestational age, crown-rump length, number and sex of the fetuses examined.

**Table 1:** Age, number and sex of the fetuses studied

Gestational age	Crown-rump length (mm)				Number of fetuses	Sex	
Weeks (Hbd-life)	Mean	SD	Min.	Max.		♂	♀
18	133.33	5.80	130.0	140.0	3	1	2
19	150.00	3.03	146.0	154.0	6	2	4
20	159.67	0.58	159.0	160.0	3	2	1
21	174.67	3.51	171.0	178.0	3	2	1
22	186.00		186.0	186.0	2	0	2
23	196.33	1.15	195.0	197.0	3	1	2
24	208.67	3.81	204.0	213.0	9	5	4
25	214.00		214.0	214.0	1	0	1
26	229.00	5.70	225.0	233.0	2	1	1
27	239.25	2.36	236.0	241.0	4	4	0
28	249.50	0.70	249.0	250.0	2	0	2
29	253.00		253.0	253.0	1	0	1
30	263.67	1.15	263.0	265.0	3	3	0
Total					42	21	21

**Fig. 1:**
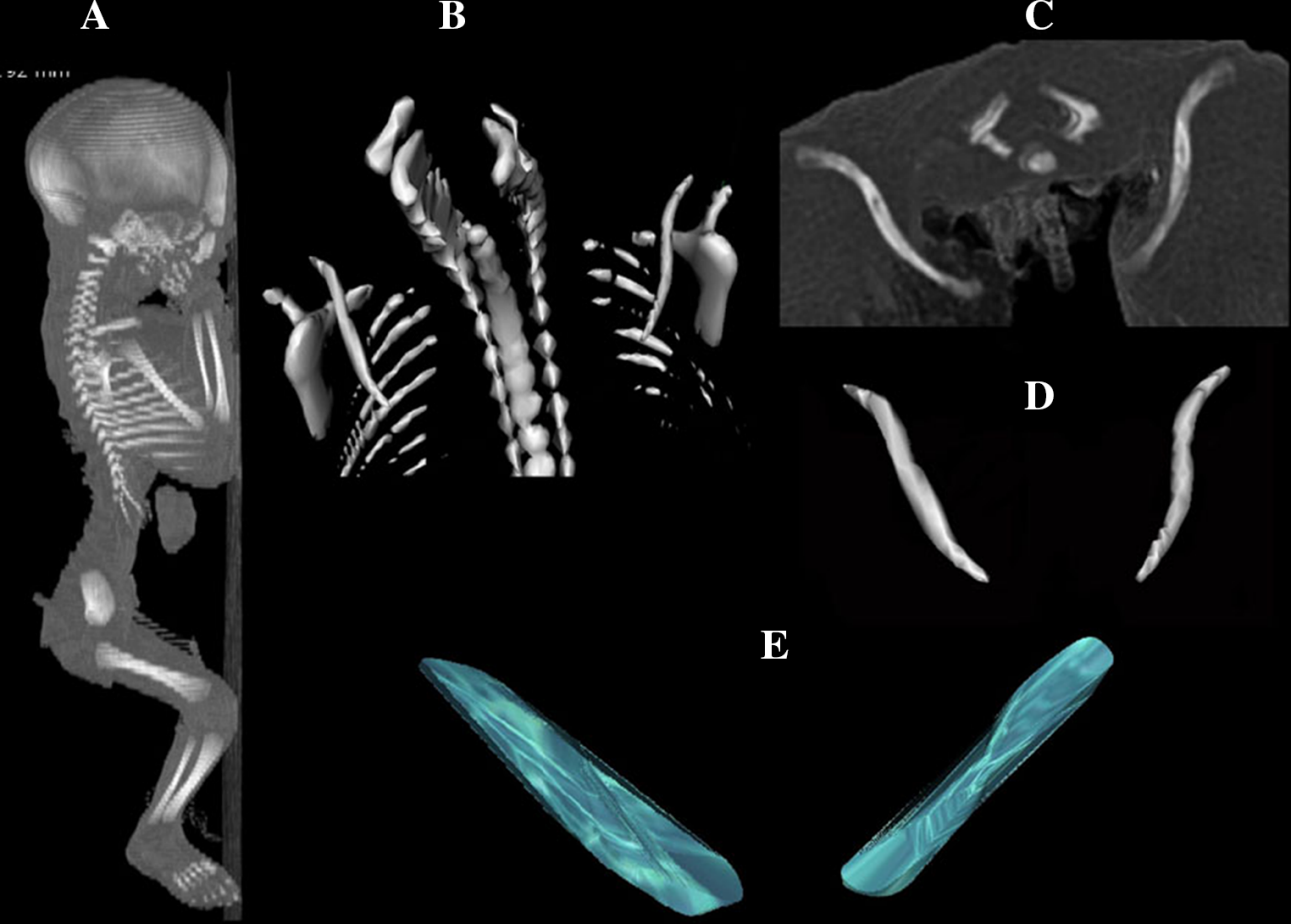
CT of a male fetus aged 26 weeks (in the sagittal projection) recorded in DICOM formats (*a*) with further reconstructions of its clavicles in superior-anterior (*b*) and horizontal (*c*, *d*) projections, including its fused primary ossification centers (*e*), assessed by Osirix 3.9

Using Siemens Biograph 128 mCT, the fetal CT scans were recorded in DICOM formats with the reconstructed slice width option of 0.4 mm (Fig. 1a). Such a technique is a prerequisite for further three-dimensional reconstructions (Fig. 1b–e) and morphometric analysis of objects given [1, 25–29]. The gray scale in Hounsfield units of achieved CT pictures ranged from −275 to −134 for a minimum, and from +1165 to +1558 for a maximum. Thus, the window width (WW) alternated from 1404 to 1692, and the window level (WL) varied from +463 to +712. In every individual, the fused ossifications centers of the right and left clavicles were measured in relation to their linear dimensions, cross-sectional areas and volumes. Although the sternal and acromial ends of the clavicles studied still remained cartilaginous, their contours could be evidently delineated [2, 4].

**Fig. 2:**
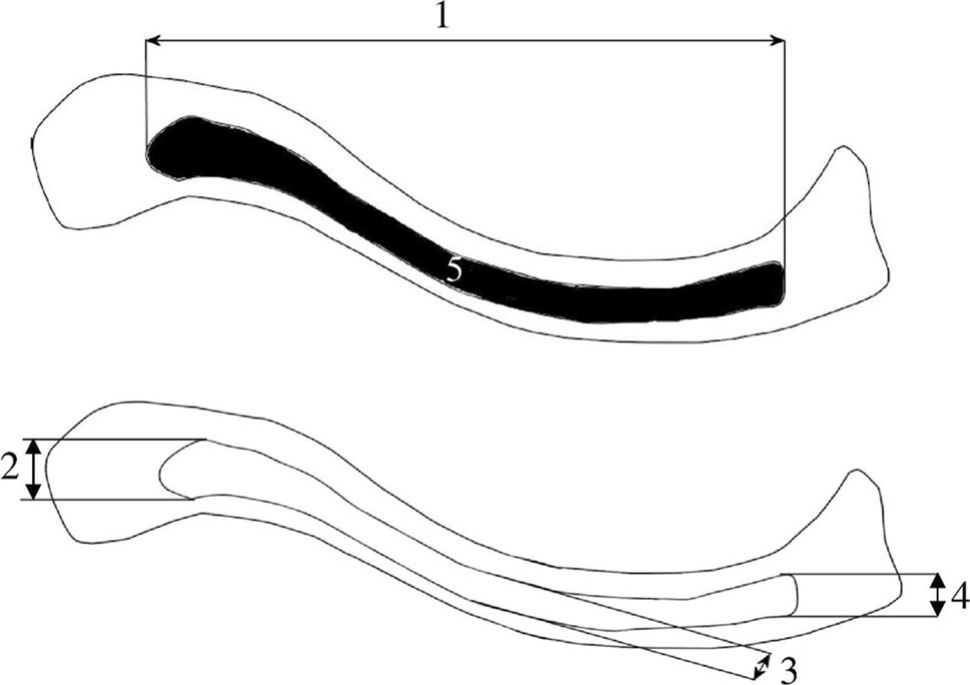
Diagram showing measurements of the fused primary ossification centers of the clavicle in the horizontal projection: *1* transverse diameter, *2* sagittal diameter of the lateral end, *3* sagittal diameter of the middle part, *4* sagittal diameter of the medial end, *5* cross-sectional area

**Fig. 3:**
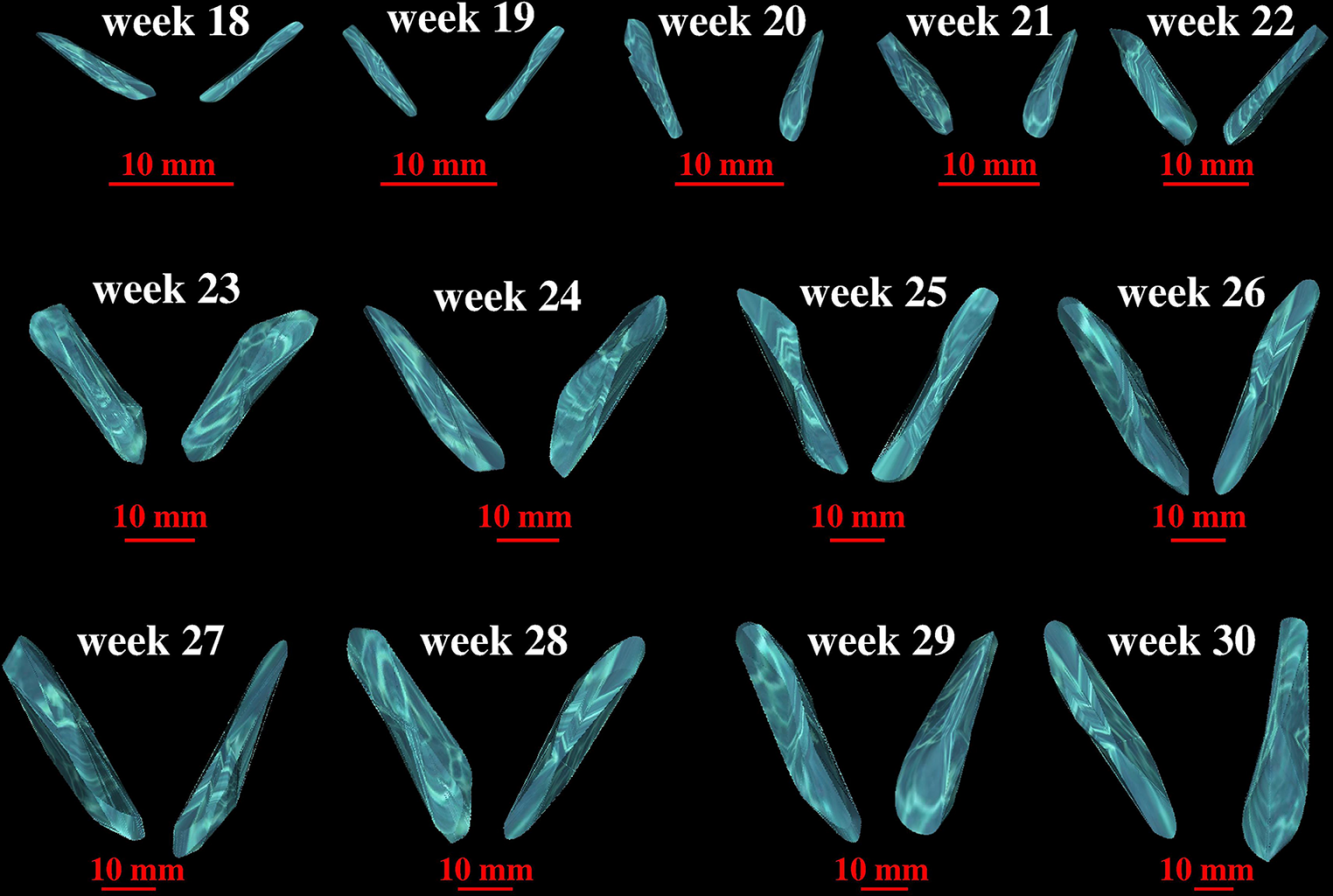
Fused primary ossification centers of the right and left clavicles in fetuses aged 18–30 weeks

For each clavicle ossification center the following five measurements in the transverse projection (Fig. 2) and one calculation (volume) were computed:

- transverse diameter in mm, corresponding to the distance between its lateral and medial borderlines,
- sagittal diameter of the lateral end in mm, corresponding to the distance between its anterior and posterior borderlines at the lateral end,
- sagittal diameter of the middle part in mm, corresponding to the distance between its anterior and posterior borderlines of midshaft,
- sagittal diameter of the medial end in mm, corresponding to its anterior and posterior borderlines at the medial end,
- cross-sectional area in mm^2^, corresponding to its total projection surface area, and
- volume in mm^3^, calculated due to advanced tri-dimensional reconstruction with the use of Osirix 3.9 (Figs. 1e, 3).

In an incessant attempt to reduce measurements and observer bias, all measurements were completed by one researcher (M.B). Each measurement was reiterated three times under the same conditions but at different times, and then averaged. The intra-observer variation was assessed by the one-way ANOVA test for paired data. The individual results obtained were subjected to statistical analysis. Normality of distribution and homogeneity of variance were verified by the Shapiro–Wilk test and Fisher’s test, respectively. Thus, our results have been presented as arithmetic means with standard deviations (SD). The statistical analysis was started by evaluating the likelihood of appearance of statistically significant differences in values in relation to sex (Student *t* test for unpaired variables) and laterality (Student *t* test for paired variables). In order to judge whether variables altered significantly with age, the one-way ANOVA test and the post hoc RIR Tukey test were used. So as to examine sex differences, we checked possible differences between the following three age groups: 18–21, 22–25 and 26–30 weeks. Furthermore, we tested sex differences for the entire examined cohort, without taking into consideration the fetal ages. Linear and curvilinear regression analysis was used to plot the best-fit curve for each parameter studied against gestational age, with assessing coefficients of determination (*R*^2^) between each parameter and gestational age. The relationship between variables was also estimated with the Pearson correlation coefficient (*r*).

## Results

Numerical data (mean ± SD) of the fused ossification centers of the right and left clavicles for their transverse and sagittal diameters are offered in Tables 2 and 3, while for their cross-sectional areas and volumes in Table 4. Since neither male–female nor right-left significant differences were found in values of the parameters studied, no attempt was made to separately model nomograms with relation to sex and laterality. By contrast, a statistically significant increase (*P* = 0.0000, the one-way ANOVA test for unpaired data and post hoc RIR Tukey test) in values of all measurements with gestational age was found.

**Table 2:** Transverse and sagittal diameters for: medial end, middle part and lateral end of the fused ossification centers in the right clavicle in human fetuses

Gestational age (weeks)	Number of fetuses	Fused ossification centers of the right clavicle							
		Transverse diameter (mm)		Sagittal diameter (mm)					
				Lateral end		Middle part		Medial end	
		Mean	SD	Mean	SD	Mean	SD	Mean	SD
18	3	13.33	0.59	1.50	0.21	1.47	0.09	1.45	0.03
19	6	13.67	1.48	1.39	0.23	1.51	0.05	1.41	0.09
20	3	14.50	0.96	1.49	0.23	1.40	0.13	1.39	0.08
21	3	16.29	2.64	1.87	0.24	1.62	0.26	1.54	0.19
		↓ (*P* < 0.01)		↓ (*P* < 0.01)		↓ (*P* < 0.05)		↓ (*P* < 0.05)	
22	2	16.41	0.01	1.78	0.01	1.84	0.01	1.42	0.01
23	3	16.20	0.10	1.92	0.36	1.55	0.05	1.63	0.29
24	9	16.05	0.93	2.33	0.14	1.81	0.23	1.88	0.27
25	1	18.41	1.00	2.11		1.92		2.17	
		↓ (*P* < 0.05)		↓ (*P* < 0.01)		↓ (*P* < 0.01)		↓ (*P* < 0.01)	
26	2	18.35	0.19	2.91	0.11	2.01	0.13	1.98	0.06
27	4	18.23	1.42	2.73	0.39	2.19	0.16	2.12	0.12
28	2	20.50	0.28	2.31	0.02	2.02	0.01	2.29	0.01
29	1	18.78		2.74		2.21		2.23	
30	3	19.87	1.08	2.64	0.31	2.22	0.14	2.21	0.17

**Table 3:** Transverse and sagittal dimensions for: medial end, middle part and lateral end of the fused ossification centres in left clavicle in human fetuses

Gestational age (weeks)	Number of fetuses	Fused ossification centers of the left clavicle							
		Transverse diameter (mm)		Sagittal diameter (mm)					
				Lateral end		Middle part		Medial end	
		Mean	SD	Mean	SD	Mean	SD	Mean	SD
18	3	12.71	0.56	1.84	0.17	1.37	0.02	1.31	0.11
19	6	12.76	1.51	1.43	0.12	1.30	0.14	1.32	0.14
20	3	14.49	0.55	1.93	0.08	1.34	0.30	1.36	0.03
21	3	14.93	1.41	1.85	0.09	1.43	0.25	1.75	0.23
		↓ (*P* < 0.01)		↓ (*P* < 0.01)		↓ (*P* < 0.01)		↓ (*P* < 0.05)	
22	2	13.94	0.01	2.25	0.02	1.77	0.01	1.51	0.01
23	3	16.58	1.06	2.16	0.41	1.73	0.32	1.82	0.36
24	9	16.64	2.22	2.40	0.26	1.96	0.25	1.84	0.26
25	1	18.03		2.21		2.11		1.78	
		↓ (*P* < 0.01)		↓ (*P* < 0.01)		↓ (*P* < 0.05)		↓ (*P* < 0.01)	
26	2	19.94	2.74	2.46	0.31	2.12	0.38	1.96	0.34
27	4	18.76	2.08	2.86	0.24	1.90	0.10	2.22	0.29
28	2	18.40	0.06	3.08	0.01	2.45	0.01	2.14	0.02
29	1	20.70		3.19		2.60		2.10	
30	3	22.10	0.90	3.03	0.15	2.49	0.25	2.56	0.17

**Table 4:** Cross-sectional area and volume of the fused ossification centers of the clavicle

Gestational age (weeks)	Number of fetuses	Fused ossification centers of clavicle							
		Cross-sectional area (mm^2^)				Volume (mm^3^)			
		Right clavicle		Left clavicle		Right clavicle		Left clavicle	
		Mean	SD	Mean	SD	Mean	SD	Mean	SD
18	3	12.71	2.39	16.47	1.17	46.44	5.15	64.37	6.71
19	6	12.13	2.04	17.21	1.89	39.58	3.30	39.93	3.83
20	3	14.87	0.58	16.21	0.69	52.74	10.27	47.84	2.89
21	3	19.93	5.29	17.50	1.61	70.30	18.21	65.87	11.44
		↓ (*P* < 0.01)		↓ (*P* < 0.01)		↓ (*P* < 0.01)		↓ (*P* < 0.01)	
22	2	22.30	0.14	22.10	0.14	54.10	0.14	66.05	7.14
23	3	21.00	4.52	25.90	4.53	69.03	13.44	65.33	20.65
24	9	24.97	3.87	25.98	6.38	85.62	16.12	87.43	16.68
25	1	34.40		26.60		80.90		88.10	
		↓ (*P* < 0.01)		↓ (*P* < 0.01)		↓ (*P* < 0.01)		↓ (*P* < 0.01)	
26	2	38.10	5.94	37.20	4.53	134.35	18.03	142.95	2.05
27	4	36.15	6.07	34.80	5.97	128.95	17.95	116.23	17.55
28	2	28.80	0.28	33.75	0.07	117.80	0.14	108.20	0.85
29	1	41.40		35.20		129.90		131.70	
30	3	39.43	8.34	45.77	2.66	152.60	10.89	152.77	21.77

**Fig. 4:**
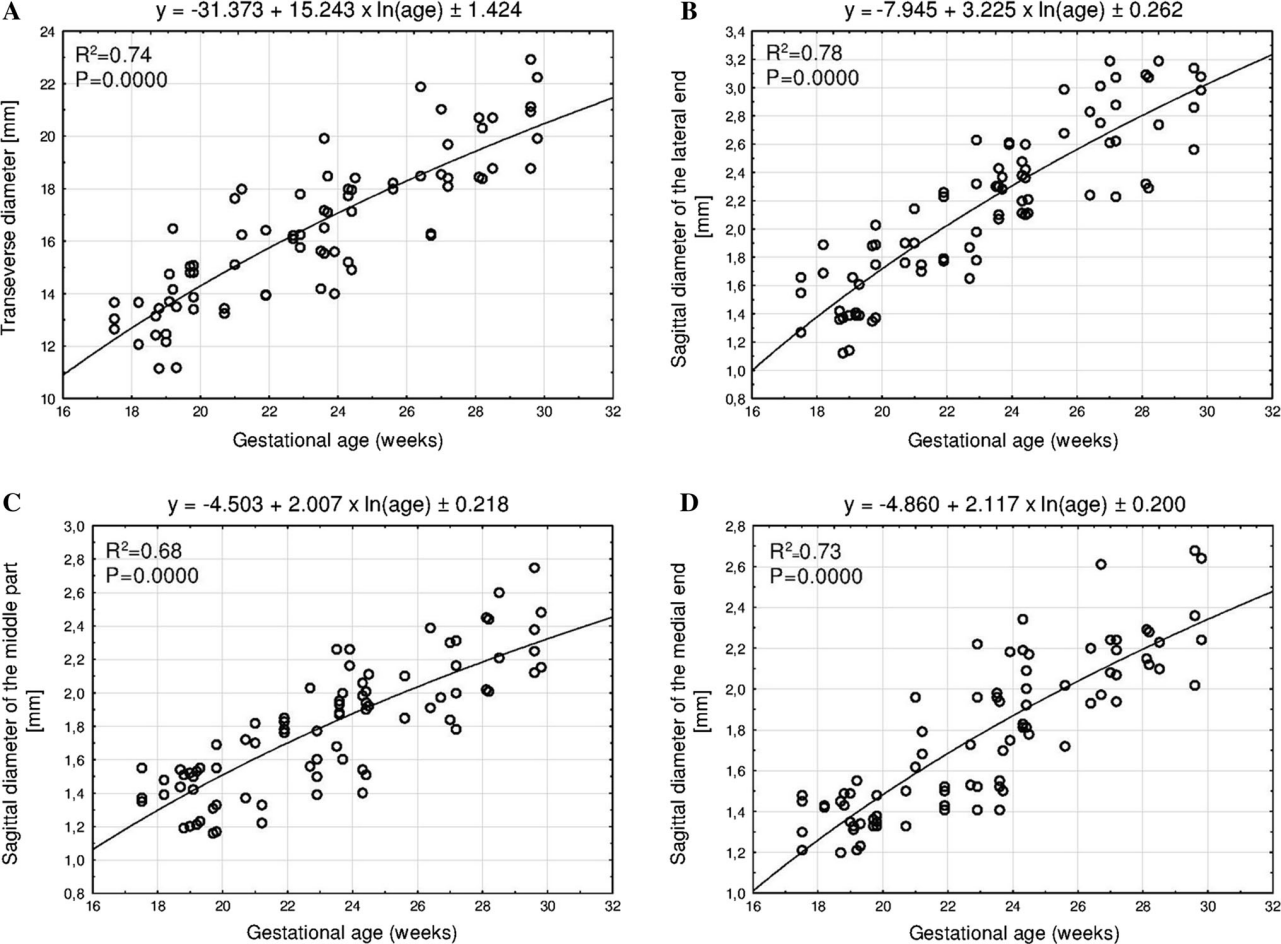
Regression lines for transverse diameter (**a**) and sagittal diameters of the lateral end (**b**), middle part (**c**), and medial end (**d**) of the fused primary ossification centers of the clavicle

The mean transverse diameter of the ossification center in the right clavicle ranged from 13.33 ± 0.59 mm at week 18 to 19.87 ± 1.08 mm at week 30. At the same time, in the left clavicle it ranged from 12.71 ± 0.56 to 22.10 ± 0.90 mm. The best fit growth model for transverse diameter (Fig. 4a) followed the natural logarithmic function *y* = −31.373 + 15.243 × ln(age) ± 1.424 (*R*^2^ = 0.74).

Between gestational ages of 18 and 30 weeks, the mean sagittal diameter of the lateral end of the ossification center changed its value from 1.50 ± 0.21 to 2.64 ± 0.31 mm, and from 1.84 ± 0.17 to 3.03 ± 0.15 mm in the right and left clavicles, respectively. In the analyzed period, the lateral end revealed a logarithmic increase in sagittal diameter (Fig. 4b), as follows *y* = −7.945 + 3.225 × ln(age) ± 0.262 (*R*^2^ = 0.78). The mean sagittal diameter of the middle part of the ossification center increased from 1.47 ± 0.09 to 2.22 ± 0.14 mm on the right, and from 1.37 ± 0.02 to 2.49 ± 0.25 mm in a 18-week fetus and a 30-week fetus, respectively. Its growth dynamics modelled the natural logarithmic function (Fig. 4c): *y* = −4.503 + 2.007 × ln(age) ± 0.218 (*R*^2^ = 0.68). The mean sagittal diameter of the medial end of the ossification center in the right clavicle ranged from 1.45 ± 0.03 to 2.21 ± 0.17 mm on the right, and from 1.31 ± 0.11 to 2.56 ± 0.17 mm in fetuses aged 18 and 30 weeks, respectively. During that period, the medial end of the ossification center increased in sagittal diameter with accordance to the natural logarithmic model (Fig. 4d): *y* = −4.860 + 2.117 × ln(age) ± 0.200 (*R*^2^ = 0.73).

**Fig. 5:**
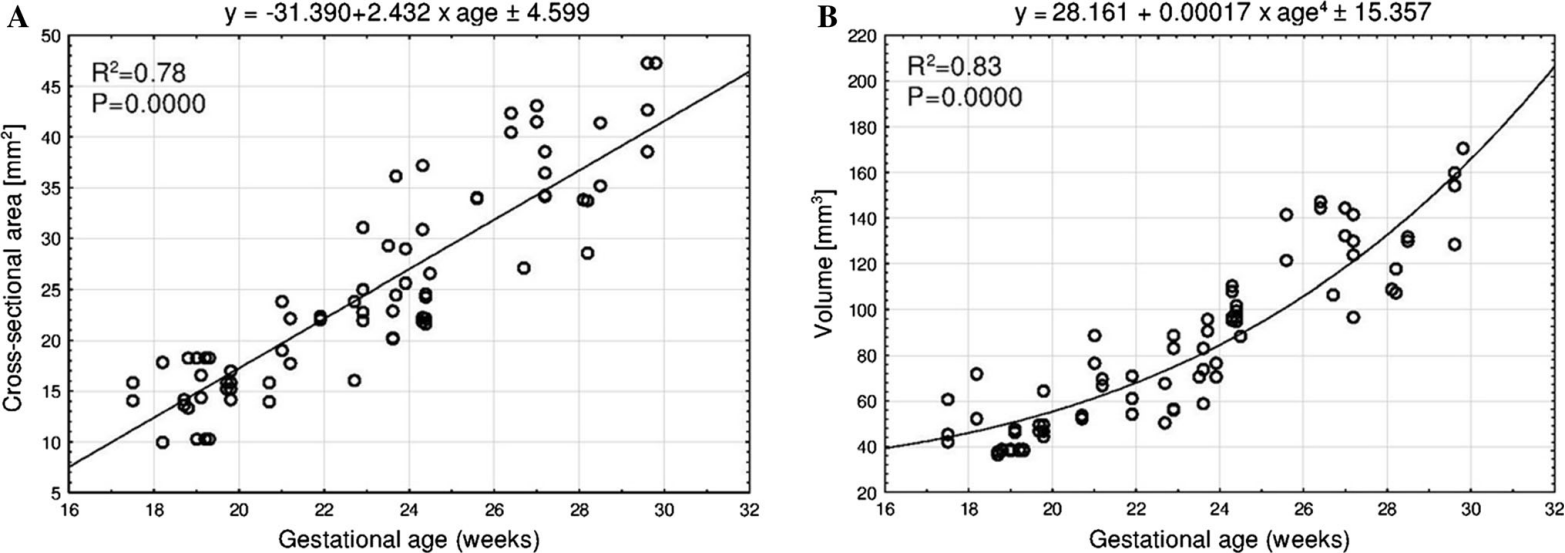
Regression lines for cross-sectional area (**a**) and volume (**b**) of the fused primary ossification centers of the clavicle

The mean value of cross-sectional area of the ossification center in the right and left clavicles grew between 18 and 30 weeks from 12.71 ± 2.39 to 39.43 ± 8.34 mm^2^, and from 16.47 ± 1.17 to 45.77 ± 2.66 mm^2^, respectively. An increase in cross-sectional area of the clavicle ossification center was typical of the linear model (Fig. 5a): *y* = −31.390 + 2.432 × age ± 4.599 (*R*^2^ = 0.78).

In fetuses aged 18 and 30 weeks, the mean volume of the right and left clavicle ossification centers raised from 46.44 ± 5.15 to 152.60 ± 10.89 mm^3^, and from 64.37 ± 6.71 to 152.77 ± 21.77 mm^3^. Thus, the volumetric growth in question generated the fourth-degree polynomial function (Fig. 5b): *y* = 28.161 + 0.00017 × age^4^ ± 15.357 (*R*^2^ = 0.83).

## Discussion

Extensive advances in medical engineering, mainly due to three-dimensional ultrasound, CT and MRI technologies are of crucial relevance in evaluating and monitoring most fetal structures [1]. These imaging techniques also facilitate to compute three-dimensional reconstructions and to analyze chosen objects, including bone structures [7]. Thus, innovative possibilities for 3-D reconstruction and volume calculation become an increasingly common approach in anatomy, clinical radiology [19, 21, 23, 36] and forensic medicine [19]. In the present study we used CT scans stored in DICOM formats that were further subjected to digital image analysis. We have been engaged on this methodology when working on the ossification process of the spine in the human fetus. Our cross-sectional study concentrated on the growth of all vertebral bodies [28], body ossification centers [29], and neural ossification centers [2, 13]. Furthermore, we completely presented the growth of three mid-point typical vertebrae, i.e. C_4_ [1], T_6_ [30], and L_3_ [27].

The clavicle in man is derived partly from both membranous and cartilaginous constituents [25]. The clavicle is the very first bone to ossify in the growing embryo, mainly by intramembranous ossification [6, 8, 10, 13, 14, 17, 30]. The presence of two conspicuously identifiable bony centers without any previous cartilaginous anlage, a larger cylindrical medial mass and a smaller flat lateral mass, in the clavicle shaft of the embryo aged 5–7 weeks (Streeter’s stages 17–19) was indubitably confirmed by numerous authors [8, 9, 14, 15, 17, 25]. After fusion of the two primary intramembranous centers by stage 20, endochondral ossification successively extends at stages 20–21 from the clavicle shaft into its cartilaginous sternal and acromial ends [9]. Obviously, a growth rate of cartilaginous sternal and acromial ends was inversely proportionate to advanced ossification of the clavicle middle part.

Findings by Ogata and Uhthoff [15] strongly supported that the site of the fused bony centers was positioned one-fourth to one-third distance from the lateral end. After fusion the bony centers, mostly the medial one, start to angulate the clavicle (stage 21) into its S-shaped appearance at 9 weeks. As reported by Ogden et al. [16] and Ogata and Uhthoff [15], the medial part contributed more to the growth in clavicle length. After that both growth and modelling of the clavicle proceed like in other long bones, i.e. by simultaneous bone formation and resorption [15]. As claimed by Fawcett [6], from a phylogenetic point of view the sternal end is older, and represents the ancestral coracoid of reptiles and birds. According to some authors [8, 17, 20, 35] there existed a highly significant difference in comparative ossification between the two sexes, with the female fetuses displaying more progressive development of ossification centers. Hypothetically, in clinical practice this fact may impede visualization of the clavicles in males during early pregnancy. However, in the material under examination we did not support a slightly more rapid rate of ossification in female fetuses than in male fetuses. Furthermore, in the material under examination there were no significant differences between numerical data for the right and left clavicles. For these reasons, our nomograms have aggregately been presented without regard to sex and laterality. The lack of sex and laterality differences in clavicle ossification centers remained in line with a histological study by Ogata and Uhthoff [15], and findings by Szymański and Kędzia [31], who analyzed radiograms showing clavicle ossification centers in human fetuses aged 16–28 weeks.

The present study is the first to provide objective information on the quantitative growth of the fused primary ossification centers of the clavicle with relation to their transverse and sagittal diameters, cross-sectional area, and volume. Objectivity of our findings results from the following three criteria: precise computerized CT DICOM images of the clavicle shaft ossification centers, clearly definite parameters, and meticulous assessment of parameters by digital image analysis of Osirix 3.9.

The results of regression analysis indicated that both the transverse and three sagittal diameters of the clavicle shaft ossification center did not reveal a proportionate growth. Instead, the best-fit growth models turned to be natural logarithmic functions: *y* = −31.373 + 15.243 × ln(age) ± 1.424 (*R*^2^ = 0.74) for its transverse diameter, *y* = −4.860 + 2.117 × ln(age) ± 0.200 (*R*^2^ = 0.73) for its sagittal diameter of the medial end, *y* = −4.503 + 2.007 × ln(age) ± 0.218 (*R*^2^ = 0.68) for its sagittal diameter of the middle part, and *y* = −7.945 + 3.225 × ln(age) ± 0.262 (*R*^2^ = 0.78) for its sagittal diameter of the lateral end. Of note, as a consequence of the four aforementioned natural logarithmic functions, their growth velocities were gradually declining with gestational age.

As far as the cross-sectional area of the ossification center is concerned, it increased proportionately, according to the model *y* = −31.390 + 2.432 × age ± 4.599 (*R*^2^ = 0.78). As claimed by Szymański and Kędzia [31], the left clavicle ossification center increased in cross-sectional area by 1.3 mm^2^ between the 4th and 5th month, by 8.4 mm^2^ between the 5th and 6th month, and by 12.9 mm^2^ between the 6th and 7th month of gestation. In turn, in the right clavicle its cross-sectional area grew by 0.2 mm^2^ between the 4th and 5th month, by 10.0 mm^2^ between the 5th and 6th month, and by 15 mm^2^ between the 6th and 7th month.

Interestingly enough, our outcomes showed that in fetuses aged 18–30 weeks the mean volume of the right and left clavicle ossification centers raised from 46.44 to 152.60 ± 10.89 mm^3^, and from 64.37 to 152.77 ± 0.1 mm^3^, with the model of choice for volume expressed as the four-degree polynomial function *y* = 28.161 + 0.00017 × age^4^ ± 15.357 (*R*^2^ = 0.74).

Unfortunately, there is no adequate quantitative information in the professional literature concerning ossification in human fetuses of different ethnic skin colors. As reported by Pryse-Davies et al. [20], no differences concerning fetuses of different ethnic skin colors reached statistical significance, though relative acceleration in the ossification of Afro-American fetuses and newborns was insinuated. On the other hand, these authors deliberated other ethnic skin variables, with increasingly advanced secondary ossification in Chinese, Malays, Indians, and Europeans, respectively. However, the lack of any numerical data obviously limits discussion on this subject.

The novelty of our study results in both numerical data and computed nomograms for the growing fused ossification centers of the clavicles in the European human fetus. This may substantially improve quantitative morphology with relation to ossification of the fetal clavicle, thereby facilitating to calculate the mean of clavicle ossification parameters according to gestational age. Our algebraic findings may be considered factual, and so relevant in the prenatal diagnosis and forensic practice, especially in monitoring normal fetal growth and screening for innate faults in fetuses suffering from cleidocranial dysplasia, thoracic outlet syndrome, congenital pseudoarthrosis of the clavicle, potential absence of the clavicle in the Abase syndrome or its incomplete ossification in individuals with trisomy 18 [3, 22, 24, 32]. Cleidocranial dysplasia presents an autosomal disorder associated with abnormal bones that usually ossify in both intramembranous and endochondral ways. It prerequisites the pathognomonic triad of malformations, i.e. (1) partial—limited to the middle and distal segments—or complete lack of the clavicles, (2) deferred closure of the frontal and occipital fontanelles, and (3) multiple excessive teeth [11, 32–34]. Thoracic outlet syndrome may result from disturbances during ossification of the clavicle shaft that are responsible for compression both the brachial plexus and subclavian artery and vein [3, 10]. Congenital pseudoarthrosis of the clavicle exists when two primary ossification centers of the clavicle failed to coalesce [24]. It is usually identified within 2 weeks after birth. Of note, congenital pseudoarthrosis of the clavicle mostly affects the right clavicle. Occasionally, pseudoarthrosis limited to the left clavicle may accompany dextrocardia or an anomalous cervical rib on the left. Maybe, this results from excessive pulsation of the subjacent subclavian artery, positioned more cephalad in the fetus than in the adult [4, 18].

## Conclusions

Neither sex nor laterality differences are found in all the studied parameters of the two fused primary ossification centers of the clavicle.

The fused primary ossification centers of the clavicle shaft grow logarithmically in both transverse and sagittal diameters, linearly in cross-sectional area, and fourth-degree polynomially in volume.

Our normative quantitative findings may be conducive in monitoring normal fetal growth and screening for inherited faults and anomalies of the clavicle in European human fetuses.

## References

[1] Baumgart M, Szpinda M, Szpinda A (2013). New anatomical data on the growing C4 vertebra and its three ossification centers in human fetuses. Surg Radiol Anat.

[2] Chano T, Matsumoto K, Ishizawa M, Morimoto S, Hukuda S, Okabe H, Kato H, Fujino S (1996). Analysis of the presence of osteocalcin, S-100 protein, and proliferating cell nuclear antigen in cells of various types of osteosarcomas. Eur J Histochem.

[3] Chen Y, Ao R, Zeng B (2009). Thoracic outlet syndrome caused by malunion of a midshaft clavicle fracture. Injury Extra.

[4] Currarino G, Herring JA (2009). Congenital pseudarthrosis of the clavicle. Pediatr Radiol.

[5] Duarte WR, Shibata T, Takenaga K, Takahashi E, Kubota K, Ohya K, Ishikawa I, Yamauchi M, Kasugai S (2003). S100A4: a novel negative regulator of mineralization and osteoblast differentiation. J Bone Miner Res.

[6] Fawcett E (1913). The development and ossification of the human clavicle. J Anat Physiol.

[7] Fiala JC (2005). Reconstruct: a free editor for serial section microscopy. J Microsc.

[8] Flecker H (1942). Time of appearance and fusion of ossification centers and observed by roentgenographic methods. Am J Roentgenol.

[9] Gardner E (1968). The embryology of the clavicle. Clin Orthop.

[10] Hansman F, Charotte MD (1962). Appearance and fusion of ossification centers in the human skeleton. Am J Roentgenol Radium Ther Nucl Med.

[11] Kumar R, Madewell JE, Swischuk LE (1989). The clavicle: normal and abnormal. Radiographics.

[12] Lam MH, Wong GY, Lao TT (2002). Reappraisal of neonatal clavicular fracture: relationship between infant size and neonatal morbidity. Am J Obstet Gynecol.

[13] Meyer DB, O’Rahilly R (1956). Roentgenographic investigation of the human skeleton during early fetal life. Am J Roentgenol Radium Ther Nucl Med.

[14] Noback CR (1954). The appearance of ossification centers and the fusion of bones. Am J Phys Anthropol.

[15] Ogata S, Uhthoff HK (1990). The early development and ossification of the human clavicle an embryologic study. Acta Orthop Scand.

[16] Ogden JA, Conlogue GJ, Bronson ML (1979). Radiology of postnatal skeletal development. III. The clavicle. Skelet Radiol.

[17] O’Rahilly R, Gardner E (1972). The initial appearance of ossification in staged human embryos. Am J Anat.

[18] Padua R, Romanini E, Conti C, Padua L, Serra F (1999). Bilateral congenital pseudoarthrosis of the clavicle report of a case with clinical, radiological and neurophysiological evaluation. Acta Orthop Belg.

[19] Page C, Taha F, Le Gars D (2002). Three-dimensional imaging of the petrous bone for the middle fossa approach to the internal acoustic meatus: an experimental study. Surg Radiol Anat.

[20] Pryse-Davies J, Smitham JH, Napier KA (1974). Factors influencing development of secondary ossification centers in the fetus and newborn. Arch Dis Child.

[21] Schmidt S, Mühler M, Schmeling A, Reisinger W, Schulz R (2007). Magnetic resonance imaging of the clavicular ossification. Int J Legal Med.

[22] Sherer DM, Sokolovski M, Dalloul M, Khoury-Collado F, Osho JA, Lamarque MD, Abulafia O (2006). Fetal clavicle length throughout gestation: a normogram. Ultrasound Obstet Gynecol.

[23] Serre T, Brunet C, Bidal S, Behr M, Ghannouchi SE, Chabert L, Durand F, Cavallero C, Bonnoit J (2002). The seated man: geometry acquisition and three-dimensional reconstruction. Surg Radiol Anat.

[24] Silveira de Figueiredo MJPS, Braga SR, Akkari M, Lopes Prado JC, Santili C (2012). Congenital pseudarthrosis of the clavicle. Rev Bras Ortop.

[25] Standring S (2008). Gray’s anatomy. The anatomical basis of clinical practice.

[26] Szpinda M, Baumgart M, Szpinda A, Woźniak A, Mila-Kierzenkowska C (2013). Cross-sectional study of the neural ossification centers of vertebrae C1–S5 in the human fetus. Surg Radiol Anat.

[27] Szpinda M, Baumgart M, Szpinda A, Woźniak A, Mila-Kierzenkowska C (2013). New patterns of the growing L3 vertebra and its 3 ossification centers in human fetuses—a CT, digital, and statistical study. Med Sci Monitor Basic Res.

[28] Szpinda M, Baumgart M, Szpinda A, Woźniak A, Mila-Kierzenkowska C (2015) Cross-sectional study of C1–S5 vertebral bodies in human fetuses. Arch Med Sci 11(1):174–189 10.5114/aoms.2013.37086 PMC4379359 25861306

[29] Szpinda M, Baumgart M, Szpinda A, Woźniak A, Małkowski B, Wiśniewski M, Mila-Kierzenkowska C, Króliczewski D (2013). Cross-sectional study of the ossification center of the C1-S5 vertebral bodies. Surg Radiol Anat.

[30] Szpinda M, Baumgart M, Szpinda A, Woźniak A, Mila-Kierzenkowska C, Dombek M, Kosiński A, Grzybiak M (2013). Morphometric study of the T6 vertebra and its three ossification centers in the human fetus. Surg Radiol Anat.

[31] Szymański M, Kędzia A (2008). Digital-image analysis of the clavicle ossification centers during prenatal period. KOWBAN.

[32] Tanaka JL, Ono E, Filho EM, Castilho JC, Moraes LC, Moraes ME (2006). Cleidocranial dysplasia: importance of radiographic images in diagnosis of the condition. J Oral Sci.

[33] Tripathi S, Singh RD, Singh SV, Chand P (2012). A case of cleidocranial dysostosis: dilemma for a prosthodontist. J Indian Prosthodont Soc..

[34] Unger S, Mornet E, Mundlos S, Blaster S, Cole DEC (2002). Severe cleidocranial dysplasia can mimic hypophosphatasia. Eur J Pediatr.

[35] Vignolo M, Ginocchio G, Parodi A, Torrisi C, Pistorio A, Venturini PL, Aicardi G, De Biasio P (2005). Fetal spine ossification: the gender and individual differences illustrated by ultrasonography. Ultrasound Med Biol.

[36] Vijayan V, El Tan C (2000) Computer-generated three-dimensional morphology of the hepatic hilar bile ducts in biliary atresia. J Pediatr Surg 35(8):1230–1235 10.1053/jpsu.2000.8760 10945701

